# How to use pace mapping for ventricular tachycardia ablation in postinfarct patients

**DOI:** 10.1111/jce.15586

**Published:** 2022-07-03

**Authors:** Charles Guenancia, Gregory Supple, Jean‐Marc Sellal, Isabelle Magnin‐Poull, Karim Benali, Nefissa Hammache, Mathieu Echivard, Francis Marchlinski, Christian de Chillou

**Affiliations:** ^1^ Cardiology Department University Hospital Dijon France; ^2^ PEC 2 EA 7460 University of Burgundy and Franche‐Comté Dijon France; ^3^ Département de Cardiologie Centre Hospitalier Universitaire (CHU de Nancy) Vandœuvre lès‐Nancy France; ^4^ INSERM‐IADI U1254 Vandœuvre lès‐Nancy France; ^5^ Division of Cardiovascular Medicine, Electrophysiology Section Hospital of the University of Pennsylvania Philadelphia Pennsylvania USA

**Keywords:** catheter ablation, electro‐anatomical mapping, ischemic cardiomyopathy, pace mapping, ventricular tachycardia

## Abstract

We aim to describe the technical aspects of pace mapping (PM), as well as the two typical patterns of pacing correlation maps during ventricular tachycardia (VT) ablation. The first main pattern is focal, with a gradual and eccentric decrease of the QRS correlation from the area with the best PM correlation. This focal pattern may be associated with two clinical situations: (1) with some endocardial points showing a good correlation compared to VT morphology: true endocardial exit of VT or endocardial breakthrough of either an intramural or an epicardial circuit; (2) without any endocardial points showing a good correlation compared to VT morphology: the VT may originate from the other ventricle, but the presence of an intramural or an epicardial circuit should be considered in patients with a structural heart disease. The second pattern is the presence of PM points exhibiting a good correlation close to other PM points showing a poor correlation compared to VT morphology: this abrupt change in paced QRS morphology over a short distance indicates divergence of activation wavefronts between these sites and suggests the presence of a slow conduction channel: the VT isthmus.

## 1 INTRODUCTION

Two main patterns are commonly found during ventricular tachycardias (VT) mapping: focal activation from a specific area of the ventricle (including automatic and micro‐reentry mechanisms) and a macro‐reentrant circuit often involving a protected isthmus of slow conduction. Pace mapping (PM) consists of stimulating the ventricular myocardium to reproduce the morphology of clinical VT on the 12‐lead ECG during sinus rhythm; it is particularly useful if VT is difficult to induce or is poorly tolerated.^1^, ^2^ Pacing that generates a QRS complex identical to that observed during an arrhythmia may allow identification of the site of origin in focal arrhythmias, whereas, in reentrant tachycardias, it implies the proximity of the exit site of the reentrant circuit. The use of PM during sinus rhythm has been well described and established as an effective technique to localize the source of focal VTs in patients with “healthy” hearts, as in those presenting with right or left ventricular outflow tract VTs.^3^, ^4^ Moreover, PM is used to identify the exit of the VT in reentrant VTs, as found in ischemic cardiomyopathies.^5^, ^6^ Critical sites of the reentry circuit and regions of slow conduction can be identified based on PM and electrogram characteristics.^7^, ^8^

## 2 TECHNICAL ASPECTS OF PM

### 2.1 Principles of PM and automated algorithms

PM can be performed at multiple sites during an EP procedure (left and right ventricles, endocardially and epicardially, according to clinical context), and the 12‐lead ECG resulting from pacing at each site is compared to that of the VT.^5^ Historically evaluated qualitatively, modern PM uses algorithms implemented in 3D mapping systems that calculate, in real time, the percentage of correlation between two ECGs. These algorithms improve the ability of PM to identify the VT sites,^9^ have a better spatial resolution than traditional ECG PM,^10^ and allow the generation of a pacing correlation map.

### 2.2 Pacing correlation map

A pacing correlation map plots the surface 12‐lead ECG morphology matching score associated with each pacing point collected compared to the originally collected template beat surface 12‐lead ECG morphology. It is available in the three main 3D mapping systems: PASO®, CARTO® (Biosense Webster); Score Map, EnSite Precision™ (Abbott); and manual annotation of the percentage of correlation in RHYTHMIA HDx™ (Boston Scientific).

**Figure 1:**
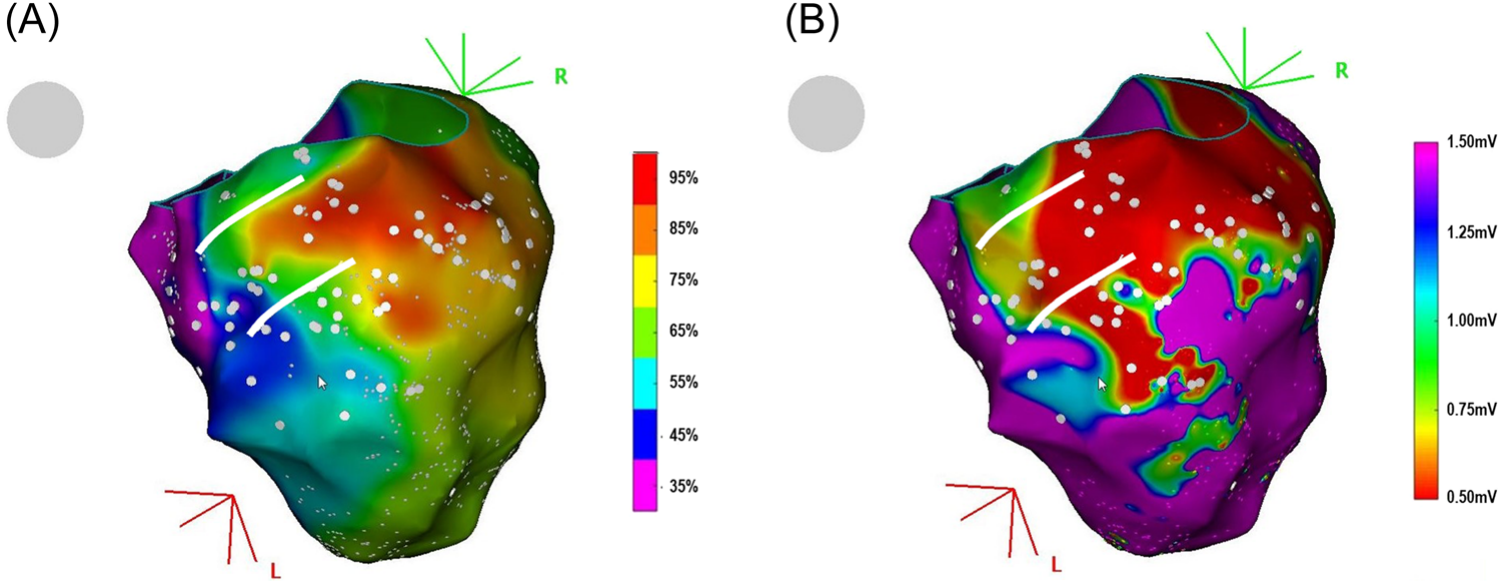
(A) Pacing correlation map of a left ventricular tachycardia on an inferior myocardial infarction scar. The color scale depicts the percentage of correlation (from 0 = purple to 100% = red) between the paced QRS and the 12‐lead reference QRS of the ventricular tachycardia. The VT isthmus borders are suggested by two parallel white lines (see further in the text for explanations on how to define isthmus borders on a pacing correlation map). White dots indicate pacing sites. (B) Bipolar left ventricular voltage map of the same clinical case.

There is no uniform definition of a “good” correlation, particularly in defining components of macro‐reentry scar‐based tachycardia, but it is estimated that at least 90% correlation is required to be considered a good correlation.^11^ Great density of sampling is often obtained in the region of interest (best correlation area): we suggest to pace at sites separated by ≦10 mm in this region of interest. As for the pacing correlation map visualization, we use the custom scale of the PASO® at the beginning of the procedure to consider all potential correlations and then adjust the scale to highlight the area of interest (Figure 1).

**Figure 2:**
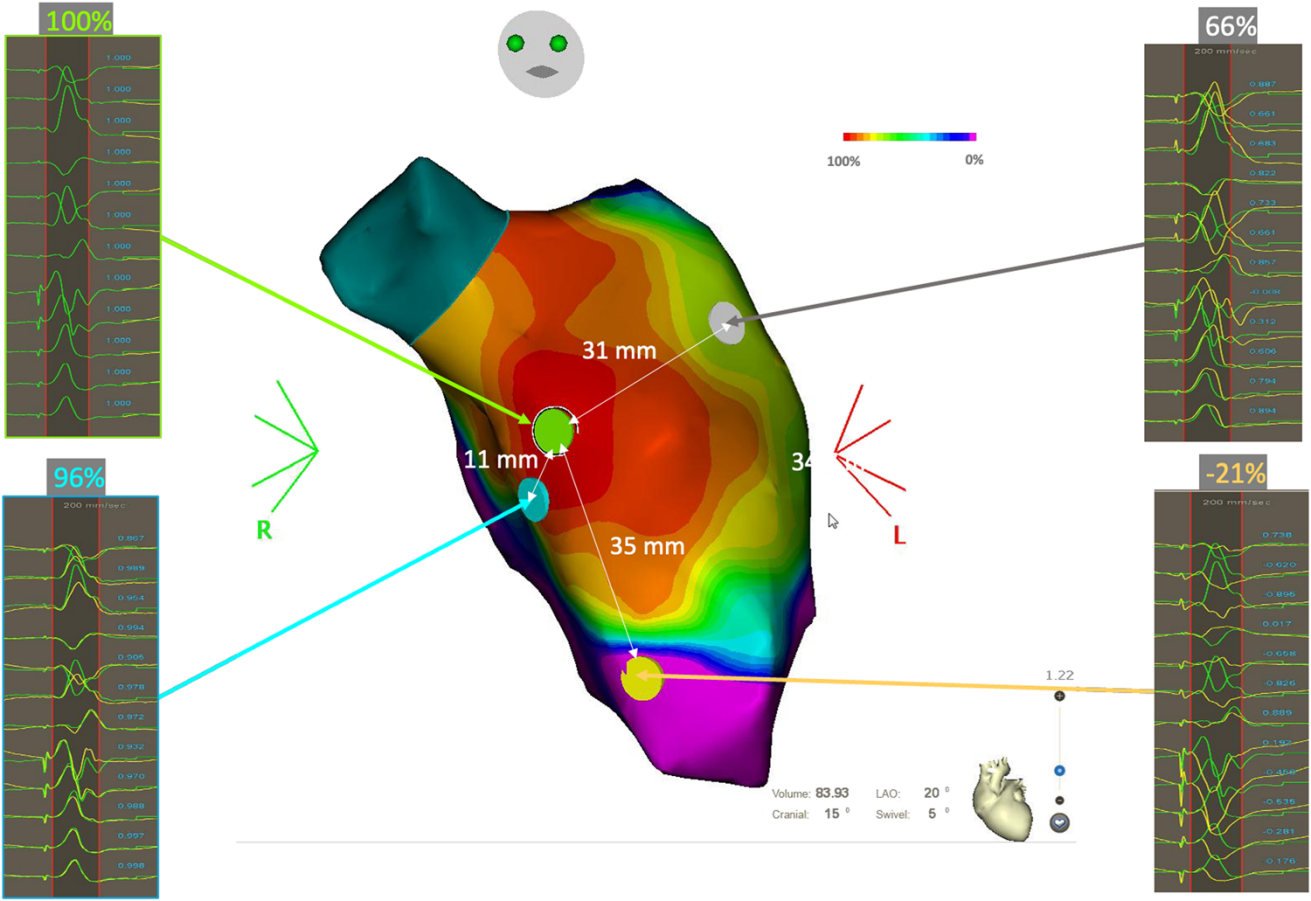
Pacing correlation map of a left ventricle during sinus rhythm. The color scale depicts the percentage of correlation (from 0 = purple to 100% = red) between the paced QRS and the reference QRS (here in sinus rhythm). The green point is taken as the pace mapping reference. In healthy tissue, pacing on sites in close proximity to each other (green and blue dots) will result in a very similar percentage correlation. The further away the pacing sites are, the more the morphology of the QRS obtained will differ (gray and yellow dots).

An important concept in PM is that a pacing correlation map can be similar to an activation map; in healthy tissue, pacing at different sites in close proximity to other will result in a very similar percentage correlation. The further away the pacing sites are, the more different the morphology of the QRS obtained will be (Figure 2). Smaller distances give better paced QRS matching and earlier activation times and vice versa for longer distances.^12^ Thus, a pacing correlation map can be analyzed as an activation map, taking into consideration the percentage of correlation of paced QRS as a function of the distance of the pacing site from the site of origin of the VT (i.e., site of earliest ventricular activation or exit), since the activation time of this point during VT is a function of the distance of this point from the site of origin of the VT^13^ (Figure 2).

### 2.3 Technical limitations of PM

Several factors impact the spatial resolution of PM maps such as pacing outputs, myocardial conduction velocities, bipolar versus unipolar pacing, or large interelectrode distance.^12^ Thus, it has been suggested to check the pacing threshold at each pacing site and conduct pacing at only two times the threshold output to avoid far‐field capture.^14^ However, we suggest a practical approach, consisting of pacing at 5 mA and adjusting upward. In our experience, the pacing mode (bipolar or unipolar) uncommonly changes the QRS morphology,^14^ so for convenience, we pace in bipolar mode, which is the default mode when the pacing channel is routed through a 3D electro‐anatomic system. Regarding catheter tip size, smaller tips allow more focal myocardial capture, improving the spatial resolution of pace maps. Finally, although some studies have suggested pacing at rates similar to VT cycle length,^15^ as the variation of coupling intervals may alter the paced QRS morphology, our unpublished data based on numerous pacing sites and on pacing rates varying from 600 to 300 ms show only a minimal and inconsistent QRS mismatch, which does not alter the reliability of the pacing correlation map in most cases. However, pacing at rapid rates may be required in selected patients but should be performed with caution given that it may be more likely to provoke arrhythmias, particularly when pacing is performed within the suspected VT circuit. Finally, a limitation of the PASO® correlation algorithm must be highlighted: it is not possible to adjust the weight of a lead of low amplitude or disturbed signal to the global correlation. This can lead to marked differences in the percentage of correlation for limited changes in QRS morphology.

## 3 TYPICAL PATTERNS OF PACING CORRELATION MAPS DURING VT ABLATION

### 3.1 Focal pattern

The first main pattern that can be found on a pacing correlation map is focal, with a gradual and eccentric decrease of the QRS correlation from the area of the best PM correlation (which does not mean a perfect PM match). This focal pattern may be associated with two very different clinical situations:

#### 3.1.1 With points exhibiting a good correlation compared to VT morphology

**Figure 3:**
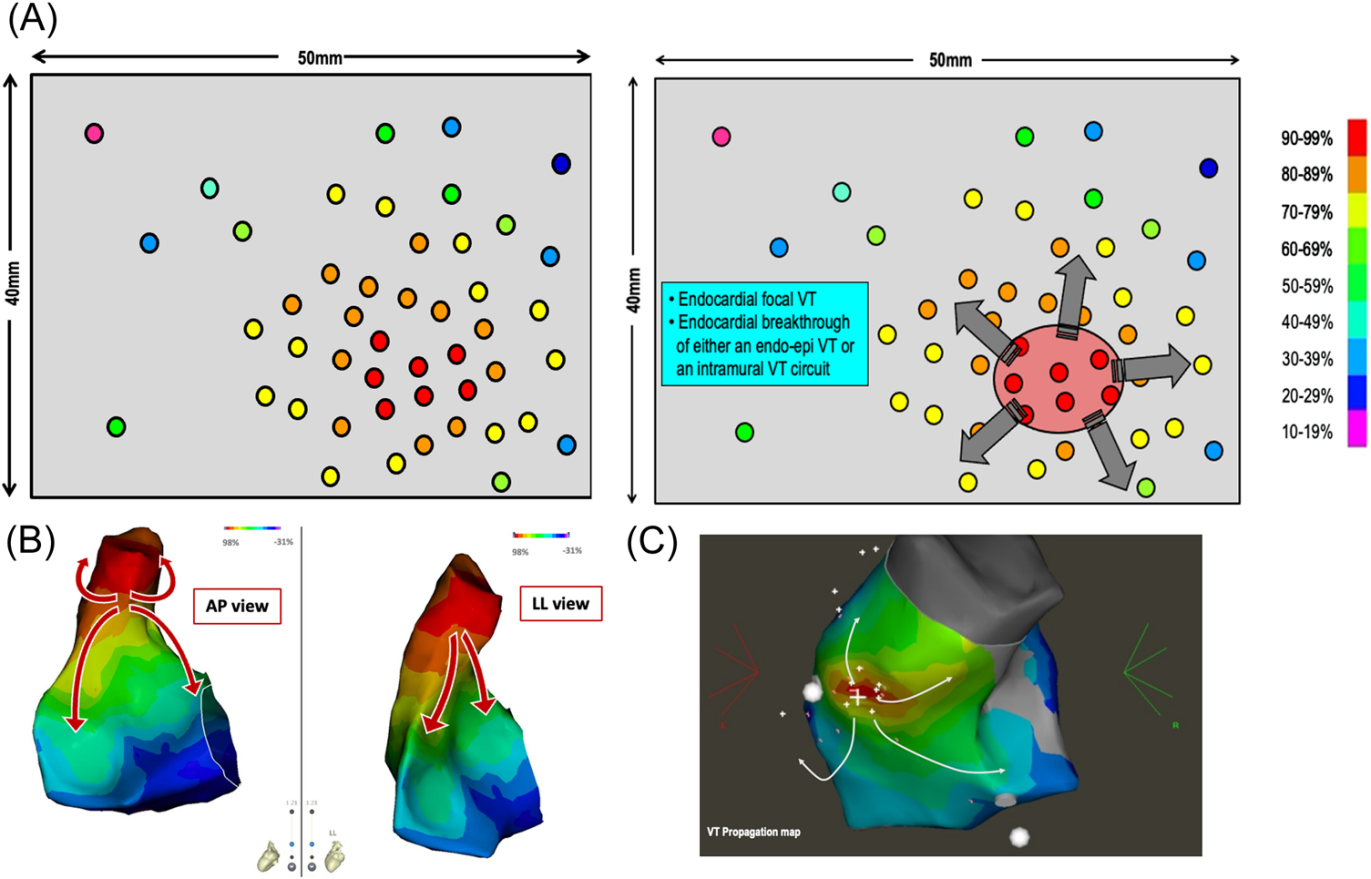
Focal pattern with points of good correlation. (A) Simulated case: the pacing correlation map shows a centrifugal pattern that suggests a focal activation pattern. Indeed, the percentage of correlation of the paced QRS is inversely proportional to the distance between the pacing site and the VT exit site. (B) Clinical case that shows a pacing correlation map in a patient with a focal ventricular tachycardia originating from the right ventricular outflow tract (RVOT). The color scale depicts the percentage of correlation between the paced QRS and the focal VT reference QRS. (C) Propagation map of a focal VT originating from the RVOT.

The first typical feature that can be observed during PM is described in Figure 3A. The rectangle represents a part of the ventricular myocardium. Pacing is performed on the endocardial surface of the myocardium. Each dot represents a paced map point and the colors of the dots correspond to the percentage of correlation with the VT. According to the color code, red dots represent a perfect match, and purple dots a poor match. The exit site of the VT is an area with a perfect match between paced and VT's QRS. In this example, the pacing correlation map shows a centrifugal pattern that suggests a focal activation pattern. Indeed, the percentage of correlation of the paced QRS is inversely proportional to the distance between the pacing site and the VT exit site. Apart from focal endocardial VTs, the same pattern can also represent an endocardial breakthrough of either an intramural or an endocardial‐to‐epicardial VT circuit. This pattern is typical in right‐ventricular outflow tract (RVOT) arrhythmias for example, but rarely found in postmyocardial infarction (MI) VTs (<5% in our experience).

Figure 3B illustrates this pattern with a clinical case. This panel shows a pacing correlation map in a patient with a focal VT originating from the RVOT. The percentage of correlation of the paced QRS gradually decreases as the pacing site moves away from the VT exit site. Figure 3C shows a propagation map during focal VT originating from the RVOT: as described in previous studies,^3^ in the case of focal VT, the “pace mapping” map also displays a centrifugal pattern. We can therefore consider that this pacing correlation map is the analog of an activation map.^16^

#### 3.1.2 Without any point showing a good correlation compared to VT morphology (the best pace mapping site does not match the VT morphology)

The second typical feature that can be observed during PM in the case of the focal pattern is illustrated in Figure 3A. Again, the pacing is performed in a given area of the myocardium, but despite the dense and homogenous distribution of pacing sites all over the endocardial surface of the ventricle, it is not possible to identify a perfect matching area. Instead, the PM map identifies an area of moderate correlation in the range of 70%−79%.

This pacing correlation map pattern represents either an epicardial VT, an intramural VT, or an endocardial to epicardial VT circuit. This pattern is found approximatively in 5%−10% of post‐MI VTs in our experience.

**Figure 4:**
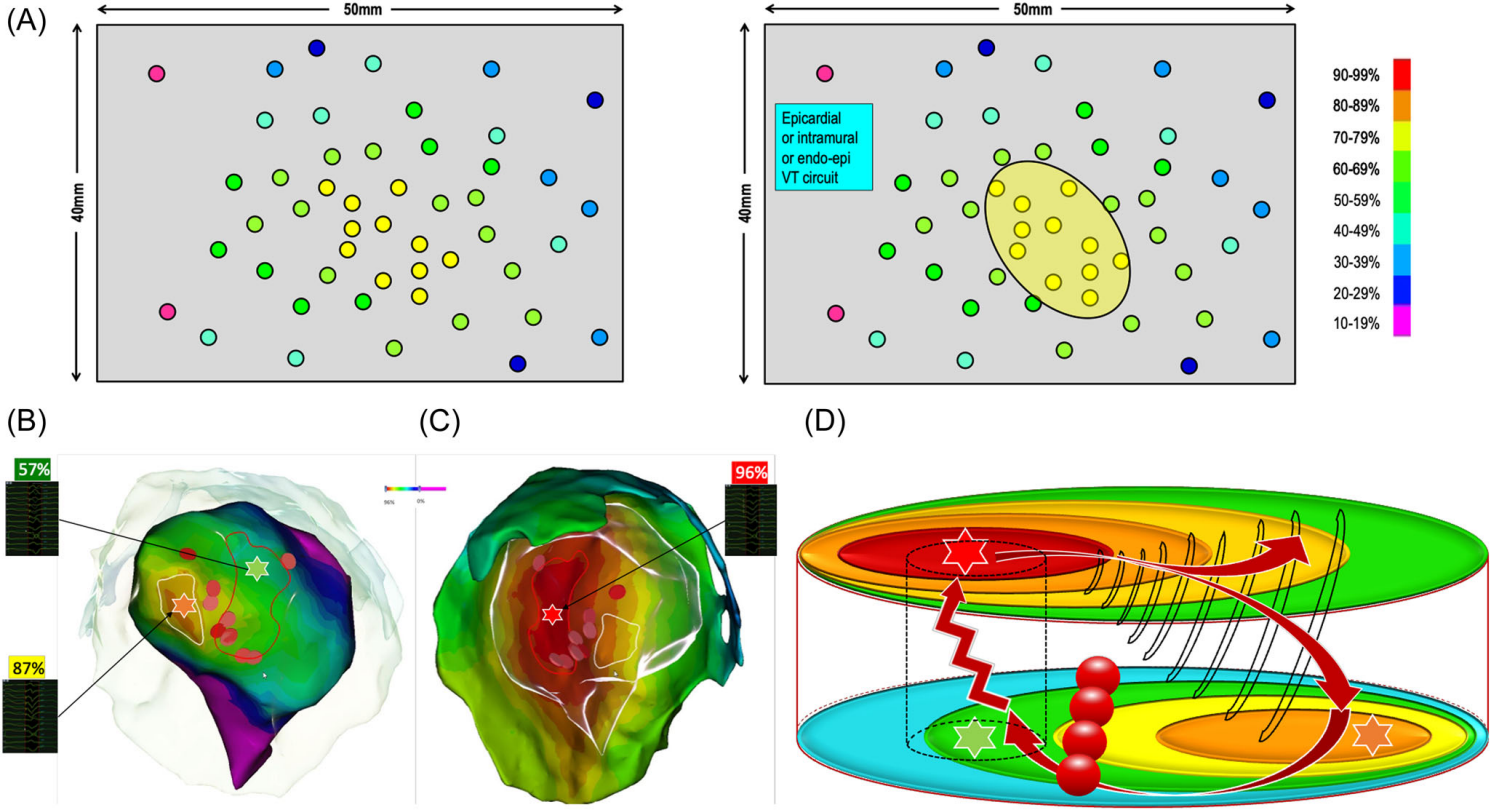
Focal pattern with missing points of good correlation. (A) Simulated case in which, despite the dense and homogenous distribution of pacing sites all over the endocardial surface of the ventricle, it is not possible to identify a perfect matching area. Instead, the PM map identifies an area of moderate correlation in the range of 70%−79%. (B) Clinical case of an endocardial left ventricular (LV) pacing correlation map. The best PM correlation point (orange star) and area (white circle) show centrifugal activation, and a poor PM correlation point (green star) is also highlighted. (C) Epicardial LV PM: a perfect match is identified on the epicardial side of the inferoapical wall (red star and red circle), with a centrifugal pacing correlation map pattern in the LV epicardium. (D) Analysis: there is a perfect match on the epicardial side of the LV (red star) and a poor match on the endocardial side (green star). Separated by a short distance, these pacing sites therefore produce ECGs showing a totally different morphology. This abrupt change in QRS morphology suggests a slow conduction zone between these pacing sites. The area highlighted by the orange star in the LV endocardium can be interpreted as the first endocardial area activated from the epicardium exit of the VT. The ablation sites are illustrated in this figure by red dots. Noninducibility was obtained after radiofrequency ablation on the endocardial entry of the VT isthmus (red dots).

Figure 4B−D illustrates this pattern, in a patient referred for postinfarct VT ablation (previous inferior myocardial infarction). In Figure 4B, a mismatch is observed at every pacing site over the entire endocardial surface of the left ventricle, with a better concordance of only 87% on the infero‐septo‐apical segment (orange star). This mismatch gradually increases when moving away from this area. The white circle shows the area of best PM points on the left ventricular endocardium, in which the percentage of correlation with the VT morphology is between 70% and 85%. This “pace mapping” map suggests that the VT exit site is not located on the endocardial surface of the left ventricle. In the absence of identification of an excellent correlation during detailed left ventricular endocardial pacing, one can consider right ventricular or epicardial pacing guided by the best correlation with pacing and assessment of the VT QRS morphology.

In this clinical case, we decided to perform epicardial mapping due to the location of the best PM points in the LV endocardium (not facing the right ventricle). A perfect match (96%) is identified on the LV epicardium (red star), with a centrifugal pacing correlation map pattern in the LV epicardium (Figure 4C). The red circle identifies the area of perfect PM correlation in the LV epicardium and is also projected on the LV endocardium views. The white circle of the “best correlation” area in the LV endocardium is projected on the LV epicardium. All PM points, in this circle, are modestly correlated with the VT morphology.

In such cases, we do not have the complete circuit on the pacing correlation map. We, therefore, have to interpret this map according to the results of the pacing sites and the presumed mechanism of VT (Figure 4D). The best correlation area is in the epicardium, showing a centrifugal decrease in the percentage of correlation with VT morphology. The exit point of the VT isthmus could be located on the LV epicardium, at the point of excellent PM (red star, 96% correlation). On the endocardial side, the best correlation area is only at 87%, lower than on the epicardial side, and also shows a centrifugal pattern of correlation decrease. The white circle presenting the “best” correlation on the LV endocardium can be interpreted as the first endocardial area activated from the epicardium exit of the VT = endocardial breakthrough of the VT. What can be inferred from these two obvious findings? The VT circuit takes a path from the epicardium to the endocardium and, given that the VT was easily inducible, reproducible, and scar related, its mechanism is most likely macro‐reentry. Taken together, these two points suggest a small reentry circuit with an epicardial exit. The path from the epicardial VT exit to the endocardial breakthrough cannot be a return path to the epicardial VT exit site. The return path (in particular the VT isthmus) must be located rather close to the epicardial breakthrough: thus the VT circuit can be either intramural with an epicardial exit or an endo‐epi reentry. We have to keep in mind that the LV wall, in postinfarct scar areas, is quite thin, usually less than 5 mm thick. When there is a point showing a poor correlation with VT morphology (green star, 57% correlation) within 5 mm of a PM point of excellent correlation, there is an abrupt change in correlation between the endocardium and the epicardium, as seen in VT isthmuses belonging to endocardial postinfarct VT circuits. However, in the absence of VT entrainment at the suspected entry site, it was not possible to exclude the possibility of an outer loop or even a bystander site. Since there is no way to explore the intramural layers, we chose a pragmatic approach and decided to apply radiofrequency energy to the suspected endocardial entrance of the VT isthmus (red dots in Figure 4B−D), resulting in noninducibility at programmed ventricular stimulation after ablation.

As mentioned before, a pacing correlation map can be analyzed as an activation map. Just following the color coding of the pacing correlation map may unmask the VT circuit. From the VT exit site, we deduced that the activation wavefront propagates centrifugally to depolarize the epicardium, crosses the LV wall to depolarize the endocardium along the path to the endocardial breakthrough site, then crosses the LV wall again to emerge, finally, at the VT exit site.

### 3.2 Abrupt transition between points with poor and excellent correlation

The second pattern that can be found on a pacing correlation map is the presence of PM points showing good correlation very close to other points exhibiting poor correlation compared to VT morphology. Based on previous works,^1^, ^17^ a difference of at least 30% points of PM correlation (if the good correlation is 95%, the poor correlation point should be <65% in most cases) between two points within 10 mm is needed to define a correlation mismatch.

**Figure 5:**
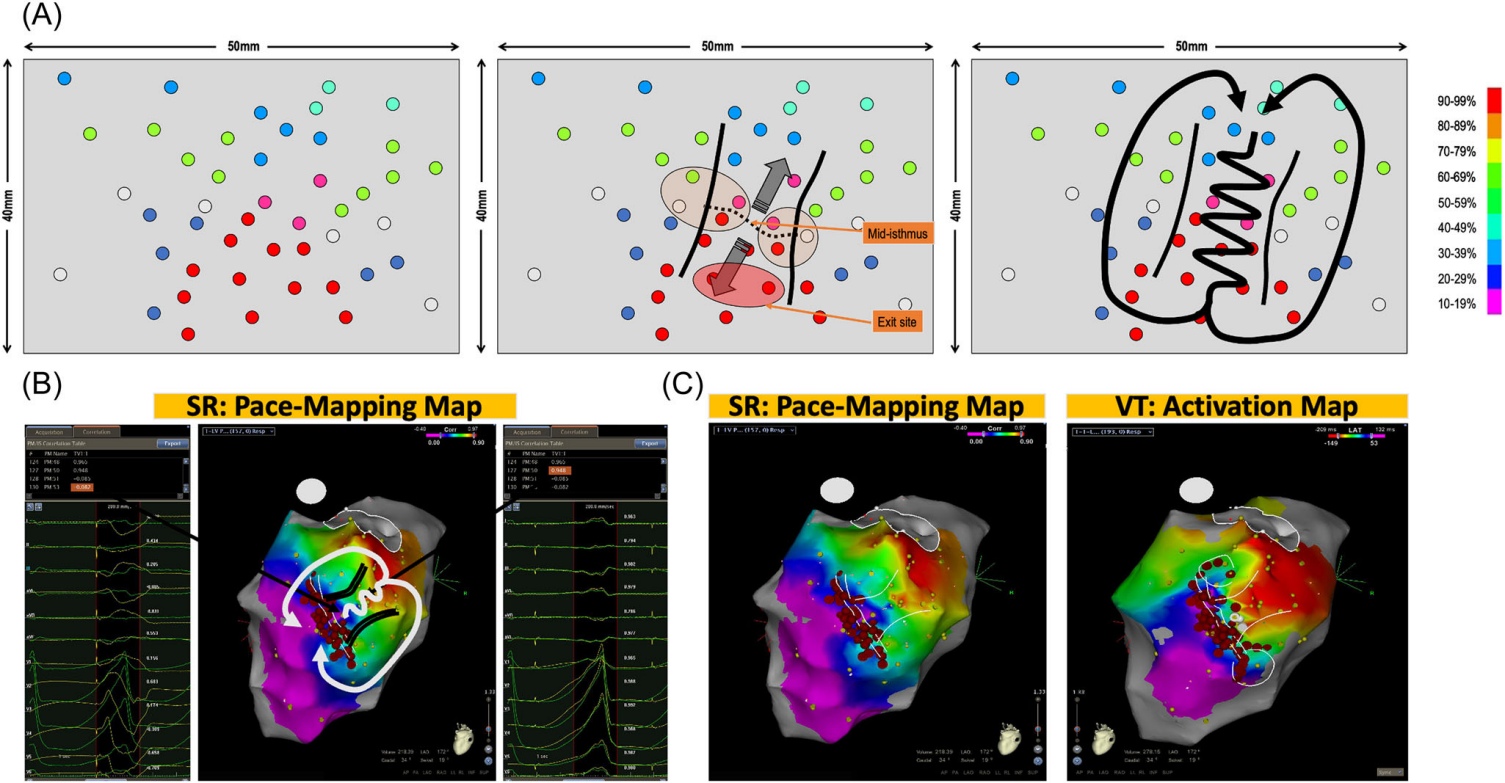
(A) Simulated case presenting the third typical feature during pace mapping: abrupt transition between points with bad and excellent correlation. In the case of points with a mismatch in the immediate vicinity of the exit zone, an area with abrupt transition is highlighted and it corresponds to the location of the VT isthmus. The virtual line, bounding the points with a perfect match from the points with a poor match, represents the mid‐isthmus line and divides the entry and exit area of the isthmus. When pacing around this area is performed, areas of intermediate matching are found laterally to the isthmus mid‐line: lateral borders of the isthmus. Finally, this pacing correlation map, as an analog of an activation map, reveals the VT circuit. (B) Clinical case presenting two pacing sites 10 mm apart. One shows a perfect match of 95% with the VT morphology and the other one shows a mismatch with a −8% concordance with the VT. This abrupt transition in QRS morphology identifies the VT isthmus and the color‐coding reveals the VT circuit. (C) In this same patient, the pacing correlation map and the VT activation map are depicted side by side and look absolutely identical.

This feature is illustrated in Figure 5A. Pacing is performed over the endocardial surface of the ventricle until an area with a perfect match is found: this area identifies the VT exit zone.^6^ Then, a denser mapping around this area is performed to determine if the pacing correlation map shows the centrifugal pattern, or not.

In the case of points with a mismatch in the immediate vicinity of the exit zone, then an area with abrupt transition is highlighted and it can correspond to the location of the VT isthmus. The virtual line, demarcating points with good PM on one side from points with a poor match on the other side, is named the “mid‐isthmus line.” This line delineates the entry and exit area of the isthmus.

If pacing around this area is performed, areas of intermediate matching will be found laterally to the mid‐isthmus line. They represent the lateral borders of the isthmus. Finally, this pacing correlation map, as would do an activation map, reveals the VT circuit. The incidence of this pattern, in postinfarct VT patients, is more than 90% in our experience.

Figure 5B depicts two pacing sites 10 mm apart. One shows a perfect match of 95% with the VT morphology and the other one shows a mismatch with a −8% concordance with the VT morphology. This abrupt transition in QRS morphology suggests the VT isthmus and the color coding reveals the VT circuit. In this same patient, depicted side by side, are the pacing correlation map and the VT activation map, which look absolutely identical (Figure 5C).

### 3.3 Technical pitfalls and limitations of pacing correlation map analysis for reentry circuits

**Figure 6:**
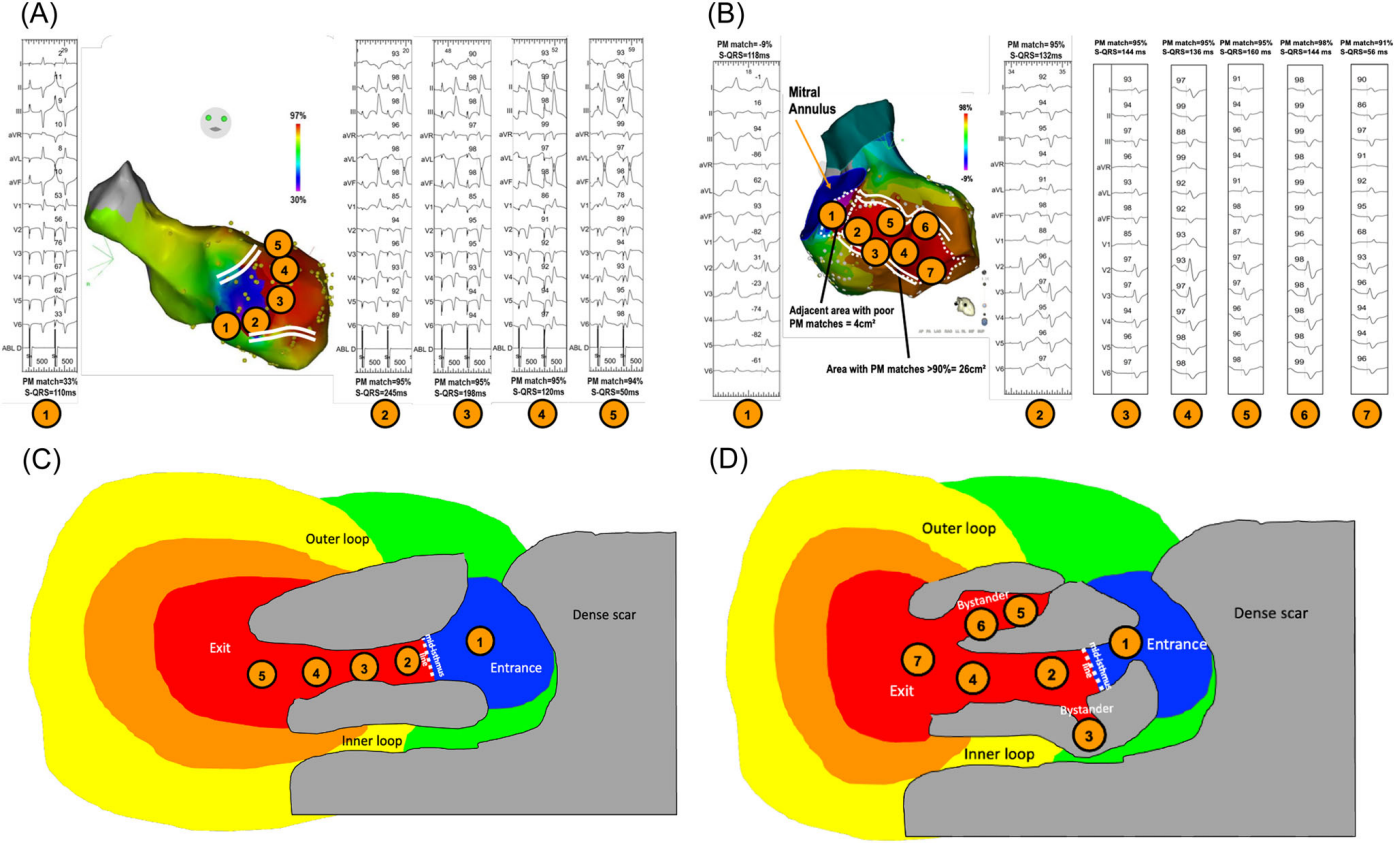
Examples of two VT circuits and the corresponding S‐QRS measurements within the “good correlation” area. Panel A shows a consistent decrease in the S‐QRS from the mid‐isthmus to the exit of the circuit, whereas Panel B shows discrepancies within a large area of good correlation (within the scar) of the S‐QRS measurements, suggesting bystander areas. Panel C illustrates Panel A map: S‐QRS duration within the area of good correlation decreases progressively along the path from the mid‐isthmus line to the exit. Panel D illustrates Panel B map: S‐QRS measurements in the area of good correlation help to discriminate between the VT isthmus and bystander areas.

**Figure 7:**
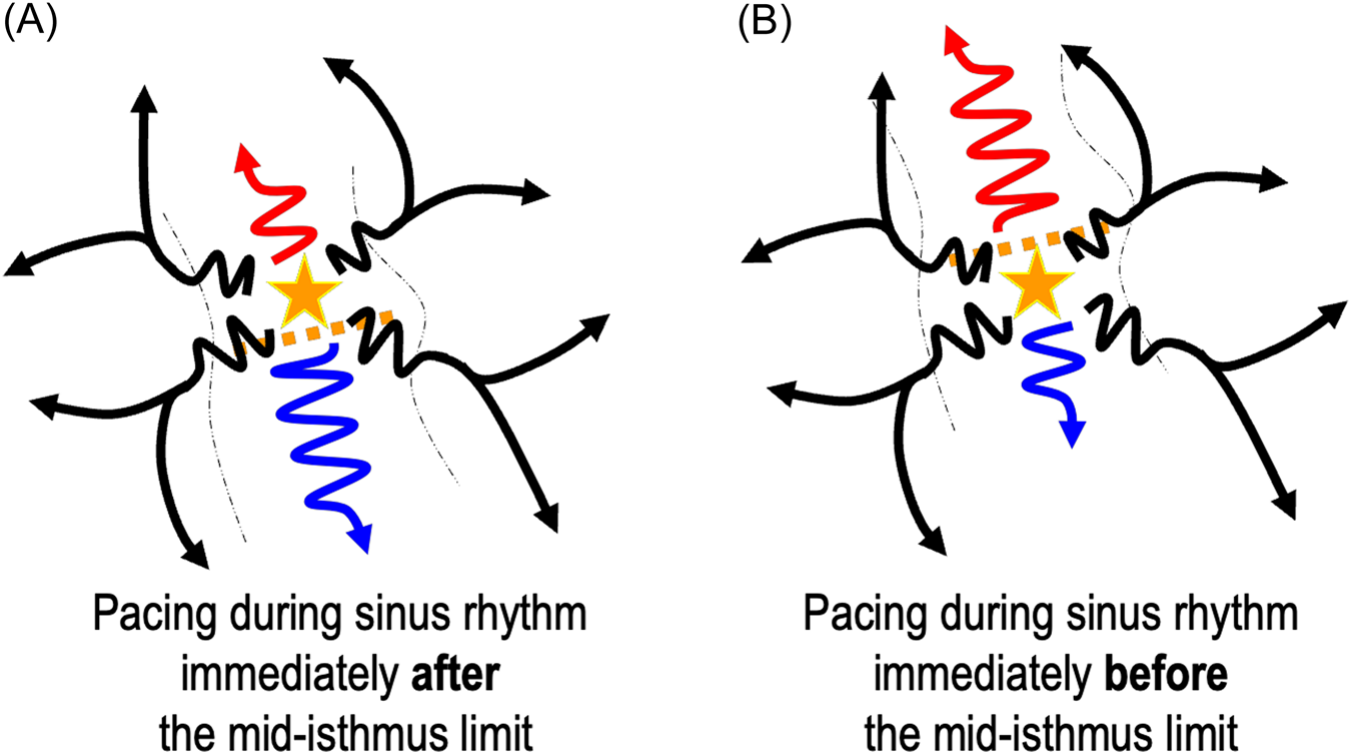
Simulated case of pacing in the VT isthmus bounded by functional barriers. Theoretically, when pacing immediately after (A) and before (B) the mid‐isthmus line, the resulting 12‐lead ECG would be very similar given the centrifugal propagation of the wavefront from the pacing site not bounded by lateral anatomical barriers. Although this has not been observed in our clinical practice, it could be a limitation of pacing correlation maps.

1. A pacing correlation map is always feasible if a VT morphology has been recorded. However, as explained in Figure 4, in the case of complex circuits involving intramural reentry, parts of the circuits are missing from the map. Thus, the VT circuit is extrapolated from the pacing correlations obtained from both sides of the myocardial layer.
2. A VT exit site may be adjacent to a scar area or a line of the block that is not involved in the VT circuit. This would create a pattern of abrupt change in pacing correlation, not related to an isthmus but rather a remote bystander area. In such cases, the pattern of the pacing correlation map would be different from that shown below and illustrated in Figures 5 and 6. Indeed, the correlation difference (or gradient) between the isthmus exit area and the outer loops of the circuit is much lower as compared to the correlation difference between the isthmus entrance and exit. In fact, when pacing at the isthmus entrance, the propagation wavefront is strictly in the opposite direction to that of the pacing in the exit area. In a previous paper comparing activation maps to pacing correlation maps,^1^ we described that these abrupt changes of correlation were consistently found within the VT isthmus and never in areas of conduction block. However, these results need to be confirmed in larger series to exclude this possibility.
3. In the case of adjacent bystanders within the VT isthmus, the paced QRS should result in the same morphology as the area of the isthmus to which it is connected, but with longer S‐QRS. Thus, this would only result in a broader isthmus on the pacing correlation map. It is important to note that the S‐QRS within the good correlation area is not always consistent with an activation pattern, progressively decreasing from the mid‐isthmus to the exit (Figure 6A−D), probably due to bystanders. As for activation maps, if possible, entrainment should be performed within the potential isthmus to confirm its involvement in the VT circuit.
4. Functional line of blocks on the isthmus borders have been described in postinfarct VT in up to one‐third of the cases.^18^, ^19^, ^20^ This could be a main limitation to pacing correlation maps given that these lines of block are not likely to be found during pace‐mapping (or activation in sinus‐rhythm). In the case of a functional block without significant conduction slowing in sinus rhythm, pacing on each side of the mid‐isthmus line would result in very similar pacing correlations and 12‐lead ECG (Figure 7). Thus, no gradient correlation area would be found within the VT isthmus. However, even if this possibility cannot be ruled out, in our experience this is not the case: the VT isthmuses identified with activation and entrainment consistently match a gradient area on the pacing‐correlation map. This could be related to the slow conduction in sinus rhythm within the area of “functional lines of blocks” in VT. It is important to note that despite the absence of an anatomical line of block during sinus rhythm, slow conduction velocities are found within these areas of the functional block as described by Anter et al.^20^ Therefore, when pacing within the VT isthmus, conduction though functional lateral boundaries could be slow enough to create a pattern of gradient correlation during pace mapping.

## 4 CONCLUSION

A pacing correlation map can mimic the activation pattern of a VT, even in macro‐reentry circuits. In the case of an endocardial “centrifugal” pacing correlation map pattern, presence of an intramural or an epicardial circuit should be considered in patients with a structural heart disease. Finally, an abrupt change in paced QRS morphology over a short distance suggests a slow conduction channel: the VT isthmus.
